# Sex differences in clinical phenotype and transitions of care among individuals dying of COVID-19 in Italy

**DOI:** 10.1186/s13293-020-00334-3

**Published:** 2020-10-16

**Authors:** Valeria Raparelli, Luigi Palmieri, Marco Canevelli, Flavia Pricci, Brigid Unim, Cinzia Lo Noce, Emanuele R. Villani, Paula A. Rochon, Louise Pilote, Nicola Vanacore, Graziano Onder, Italian National Institute of Health COVID-19 Mortality Group, Luigi Palmieri, Elvira Agazio, Xanthi Andrianou, Pierfrancesco Barbariol, Antonino Bella, Stefania Bellino, Eva Benelli, Luigi Bertinato, Stefano Boros, Gianfranco Brambilla, Giovanni Calcagnini, Marco Canevelli, Maria Rita Castrucci, Federica Censi, Alessandra Ciervo, Elisa Colaizzo, Fortunato D’Ancona, Martina Del Manso, Chiara Donfrancesco, Massimo Fabiani, Francesco Facchiano, Antonietta Filia, Marco Floridia, Fabio Galati, Marina Giuliano, Tiziana Grisetti, Yllka Kodra, Martin Langer, Ilaria Lega, Cinzia Lo Noce, Pietro Maiozzi, Fiorella Malchiodi Albedi, Valerio Manno, Margherita Martini, Alberto Mateo Urdiales, Eugenio Mattei, Claudia Meduri, Paola Meli, Giada Minelli, Manuela Nebuloni, Lorenza Nistico, Marino Nonis, Graziano Onder, Lucia Palmisano, Nicola Petrosillo, Patrizio Pezzotti, Flavia Pricci, Ornella Punzo, Vincenzo Puro, Valeria Raparelli, Giovanni Rezza, Flavia Riccardo, Maria Cristina Rota, Paolo Salerno, Debora Serra, Andrea Siddu, Paola Stefanelli, Manuela Tamburo De Bella, Dorina Tiple, Brigid Unim, Luana Vaianella, Nicola Vanacore, Monica Vichi, Emanuele Rocco Villani, Amerigo Zona, Silvio Brusaferro

**Affiliations:** 1 Department of Experimental Medicine, Sapienza University of Rome, Viale Regina Elena 324, 00161 Rome, Italy; 2 Department of Cardiovascular, Endocrine-metabolic Diseases and Aging, Istituto Superiore di Sanità, Via Giano della Bella, 34, 00161 Rome, Italy; 3 National Center for Disease Prevention and Health Promotion, Istituto Superiore di Sanità, Via Giano della Bella, 34, 00161 Rome, Italy; 4 Department of Human Neuroscience, Sapienza University of Rome, Rome, Italy; 5 Catholic University of the Sacred Heart, Largo Francesco Vito, 1, 00168 Rome, Italy; 6 Women’s College Research Institute, Women’s College Hospital, University of Toronto, 76 Grenville St, Toronto, ON M5G 1N8 Canada; 7 Centre for Outcomes Research and Evaluation, McGill University Health Centre Research Institute, 1001 boul. Décarie, Montreal, Quebec H4A 3J1 Canada

**Keywords:** Sex, COVID-19, Transition of care, Comorbidities, In-hospital complications

## Abstract

**Background:**

Among the unknowns posed by the coronavirus disease 2019 (COVID-19) outbreak, the role of biological sex to explain disease susceptibility and progression is still a matter of debate, with limited sex-disaggregated data available.

**Methods:**

A retrospective analysis was performed to assess if sex differences exist in the clinical manifestations and transitions of care among hospitalized individuals dying with laboratory-confirmed SARS-CoV-2 infection in Italy (February 27–June 11, 2020). Clinical characteristics and the times from symptoms’ onset to admission, nasopharyngeal swab, and death were compared between sexes. Adjusted multivariate analysis was performed to identify the clinical features associated with male sex.

**Results:**

Of the 32,938 COVID-19-related deaths that occurred in Italy, 3517 hospitalized and deceased individuals with COVID-19 (mean 78 ± 12 years, 33% women) were analyzed. At admission, men had a higher prevalence of ischemic heart disease (adj-OR = 1.76, 95% CI 1.39–2.23), chronic obstructive pulmonary disease (adj-OR = 1.7, 95% CI 1.29–2.27), and chronic kidney disease (adj-OR = 1.48, 95% CI 1.13–1.96), while women were older and more likely to have dementia (adj-OR = 0.73, 95% CI 0.55–0.95) and autoimmune diseases (adj-OR = 0.40, 95% CI 0.25–0.63), yet both sexes had a high level of multimorbidity. The times from symptoms’ onset to admission and nasopharyngeal swab were slightly longer in men despite a typical acute respiratory illness with more frequent fever at the onset. Men received more often experimental therapy (adj-OR = 2.89, 95% CI 1.45–5.74) and experienced more likely acute kidney injury (adj-OR = 1.47, 95% CI 1.13–1.90).

**Conclusions:**

Men and women dying with COVID-19 had different clinical manifestations and transitions of care. Identifying sex-specific features in individuals with COVID-19 and fatal outcome might inform preventive strategies.

## Introduction

The surge of the coronavirus disease 2019 (COVID-19) outbreak, caused by severe acute respiratory syndrome coronavirus 2 (SARS-CoV-2), is straining healthcare providers and researchers as they handle unprecedented unknowns. The extent to which sex, as a biological attribute, is playing a role in shaping the spreading and clinical course of the disease remains to be fully explored [1–5].

In previous coronavirus epidemics (i.e., SARS and Middle East respiratory syndrome), male sex was associated with higher vulnerability to the infection and worse outcomes [6–9]. The male predominance in mortality has emerged also in early data from the COVID-19 pandemic [4]. Differences in the immune system’s response to infection and the likelihood of comorbidities and sociocultural exposure have been pointed out as major contributing factors [4, 5]. Although the SARS-CoV-2 appears to be infecting similar numbers of women and men, case fatality rates are greater for men than women among all age groups [4, 10]. Of note, the worse outcomes in men are largely consistent across countries, with few exceptions [4, 10].

The reasons behind the male to female case fatality ratio remains mostly unknown. Overall, among the leading factors reported to strongly influence the risk of dying from COVID-19, older age (older than 65 years) and the presence of comorbidities, such as cardiovascular disease and chronic obstructive pulmonary disease (COPD) have emerged [11, 12]. Nevertheless, sex-specific data regarding the distribution of comorbidities, the clinical manifestations at presentation, and the transitions of care among individuals with severe disease are needed for better understanding of the disease and tailoring of prevention strategies. These data are often missing [13, 14].

As part of the surveillance activity of the Italian National Institute of Health (Istituto Superiore di Sanità—ISS), a sex-stratified analysis was performed with the aim to look for differences in the presentation and clinical course among women and men who died with COVID-19 in Italy.

## Methods

### Study population

In the context of the integrated system to inform and guide the Italian government’s decisions for the management of the COVID-19 outbreak, the ISS established a nation-wide surveillance network (including all the 19 Italian regions and the 2 autonomous provinces of Trento and Bolzano) aimed at collecting data from all individuals with COVID-19 throughout the country.

Medical charts from individuals with RT-PCR-confirmed SARS-CoV-2 infection who died during an in-hospital stay in Italy were analyzed. Among 32,938 COVID-19-related deaths occurred in Italy as of June 11, 2020, of whom COVID-19 was the cause directly leading to death (i.e., the underlying cause) in 89% of cases [15], 3517 medical charts (i.e., 10.7% of COVID-19-related deaths) were centrally reviewed at the ISS. These charts represented a random sample that was representative of the age, sex, and geographical distribution of all COVID-19-related deaths in the country.

On February 27, 2020, the Italian Presidency of the Council of Ministers (IPCM) authorized the collection and scientific dissemination of data concerning the COVID-19 epidemics by the ISS and other public health institutions. Therefore, ethical review and approval was not required for the study on human participants in accordance with the local legislation and institutional requirements (IPCM decree no. 620, 02-27-2020) [16].

### Data collection and measures

For in-hospital deaths related to COVID-19, medical charts were centrally analyzed. Data on demographics (i.e., age, sex, geographical macro-area) and medical history (including the presence of main pre-admission comorbidities such as ischemic heart disease [IHD], atrial fibrillation, obesity, hypertension, type 2 diabetes mellitus, COPD, congestive heart failure, [CHF], chronic kidney disease [CKD], chronic liver disease [CLD], dementia, active cancer, and autoimmune diseases) were collected. Information on COVID-19-related symptoms at admission (i.e., fever, shortness of breath [SOB], cough, diarrhea, and hemoptysis), in-hospital pharmacological treatments (i.e., antibiotics, antivirals, hydroxychloroquine, steroids, and tocilizumab) and the occurrence of complications (i.e., acute respiratory distress syndrome [ARDS], acute kidney injury [AKI], myocardial injury, shock, and co-infections) during the hospital stay were extracted, when available. Three geographical macro-areas were defined according to COVID-19 spreading in Italy: high incidence areas with sustained local transmission (mainly in the north), low incidence areas with limited but growing numbers of locally acquired cases of infection and intermediate incidence areas [17].

The transition of care of symptomatic patients was evaluated in terms of times from symptoms’ onset to (i) hospital admission, (ii) nasopharyngeal swab, (iii) intensive care unit (ICU) admission, and (iv) death. Information on the rate of admission to ICU and on the length of in-hospital stay was also collected from medical charts, if available.

### Statistical analysis

Variables found to be normally distributed were reported as mean ± standard deviation (SD), whereas those found to be non-normally distributed were reported as median and interquartile range (IQR). Differences between the groups (men vs. women) were established with the Student’s *t* test and with the Mann-Whitney *U* test, where appropriate. Categorical variables were reported as counts and percentages, and differences between groups (men vs. women) were evaluated with chi-square test or Fisher’s exact test when appropriate. Missing data were reported and ranged from 0.2 (geographical macro-area) to 21% (time to swab testing).

A logistic regression model was used to identify clinical factors and the features of the in-hospital care received associated with the male sex. Variables that significantly differed at baseline between men and women with a *p* value < 0.10 were included in the final multivariate model. A two-sided *p* value < 0.05 was considered statistically significant.

All analyses were performed using SPSS v. 26.0 (IBM, NY, USA).

## Results

Among 3517 deceased individuals with COVID-19 (mean 78 ± 12 years), 33% were women. The baseline demographic and clinical characteristics of individuals at the time of hospital admission are reported in Table 1. The prevalence of men and women hospitalized and deceased was similar across the 3 geographical macro-areas of spreading. Despite the burden of multimorbidity being similar in both women and men, women with COVID-19 were older and more likely to have CHF, dementia, and autoimmune diseases compared to men. In contrast, comorbidities such as IHD, COPD, CLD and CKD were more common in men than women.

**Table 1:** Clinical characteristics at hospital admission of COVID-19 dying individuals according to sex

	Overall (***n*** = 3517)	Women (***n*** = 1171)	Men (***n*** = 2346)	***p*** value
Age, years (mean ± SD)	77.64 **±** 11.51	80.40 **±** 11.41	76.26 **±** 11.32	< 0.001
**Age tertiles,** ***n*** **(%)**				
1° (age < 75 years)	1210 (34.4)	300 (25.6)	910 (38.8)	< 0.001
2° (75–84 years)	1285 (36.5)	383 (32.7)	902 (38.4)	
3° (> 84 years)	1022 (29.1)	488 (41.7)	534 (22.8)	
**Area of COVID-19 diffusion,** ***n*** **(%)**^b^				
High	2547 (72.6)	826 (70.8)	1721 (73.5)	0.23
Intermediate	790 (22.5)	279 (23.9)	511 (21.8)	
Low	172 (4.9)	62 (5.3)	110 (4.7)	
**Comorbidities,** ***n*** **(%)**^a^				
Ischemic heart disease	957 (27.8)	236 (20.8)	721 (31.3)	< 0.001
Atrial fibrillation	758 (22.0)	260 (22.9)	498 (21.6)	0.43
Congestive heart failure	539 (15.7)	209 (17.8)	330 (14.1)	0.002
Stroke	349 (10.2)	118 (10.4)	231 (10.0)	0.76
Hypertension	2305 (67.0)	774 (68.1)	1531 (66.5)	0.37
Type-2 diabetes mellitus	1040 (30.3)	322 (28.3)	718 (31.2)	0.09
Dementia	562 (16.3)	266 (23.4)	296 (12.9)	< 0.001
Chronic obstructive pulmonary disease	576 (16.8)	143 (12.6)	433 (18.8)	< 0.001
Active cancer	551 (16.0)	185 (16.3)	366 (15.9)	0.80
Chronic liver disease	148 (4.3)	37 (3.3)	111 (4.8)	0.032
Chronic renal disease	688 (20.0)	200 (17.6)	488 (21.2)	0.013
Dialysis	67 (1.9)	19 (1.7)	48 (2.1)	0.43
Respiratory failure	180 (5.2)	61 (5.4)	119 (5.2)	0.81
HIV	7 (0.2)	0 (0.0)	7 (0.3)	0.10
Autoimmune disease	137 (4.0)	67 (5.9)	70 (3.0)	< 0.001
Obesity	377 (11.0)	127 (11.2)	250 (10.9)	0.82
**No. of comorbidities, n (%)**				
0	144 (4.2)	33 (2.9)	111 (4.8)	0.54
1	505 (14.7)	161 (14.2)	344 (15.0)	
2	738 (21.5)	250 (22.0)	488 (21.2)	
≥ 3	2051 (59.6)	693 (60.9)	1358 (59.0)	

^a^Missing data for 79 patients (2.2%)

^b^Missing data for 8 patients (0.2%)

The clinical presentation and the transitions of care from symptoms’ onset to death are summarized in Table 2. Compared with men, women were less likely to exhibit fever, SOB, and cough as symptoms at admission. Median time from onset of symptoms to hospital admission, testing with nasopharyngeal swab, ICU admission, and death were 4 [IQR, 2–7], 5 [IQR, 3–9], 8 [IQR, 5–12], and 11 [IQR, 7–17] days, respectively. Men were more likely to experience delays in the admission to hospital and the in-hospital nasopharyngeal swab testing as compared with women. Among the 20.5% individuals admitted to ICU, men were more represented than women, but no sex differences in the time to ICU admission were observed. During the hospital stay, a higher proportion of men than women developed complications such as AKI, acute myocardial injury, and shock, while the rates of ARDS and co-infections were similar between sexes. Among the experimental pharmacological therapies, women were less frequently treated with antiviral agents (including hydroxychloroquine) and tocilizumab than men.

**Table 2:** In-hospital care and complications among dying individuals with COVID-19 according to sex

	Overall (***n*** = 3517)	Women (***n*** = 1171)	Men (***n*** = 2346)	***p*** value
**Symptoms at admission,** ***n*** **(%)**^a^				
Fever	2585 (76.3)	795 (71.3)	1790 (78.8)	< 0.001
Shortness of breath	2491 (73.6)	794 (71.2)	1697 (74.7)	0.031
Cough	1315 (38.8)	402 (36.1)	913 (40.2)	0.020
Diarrhea	193 (5.7)	72 (6.5)	121 (5.3)	0.63
Hemoptysis	19 (0.6)	5 (0.4)	14 (0.6)	0.18
**Time (in days) from symptoms’ onset to; median [IQR]**^b^				
Hospital admission	4 [2–7]	4 [2–7]	5 [2–7]	< 0.001
In-hospital nasopharyngeal swab	5 [3–9]	5 [2–9]	6 [3–9]	0.007
ICU	8 [5–12]	7 [4–12]	8 [5–12]	0.12
In-hospital death	11 [7–17]	11 [7–16]	11 [7–17]	0.06
**Length of stay (days), median [IQR]**^c^	6 [3–11]	6 [3–11]	6 [3–11]	0.80
**Admission in ICU**^d^	667 (20.5)	149 (13.9)	518 (23.8)	< 0.001
**In-hospital complications**^e^				
Acute respiratory distress syndrome	3248 (96.9)	1077 (96.4)	2171 (97.2)	0.24
Acute renal injury	741 (22.1)	187 (16.7)	554 (24.8)	< 0.001
Acute cardiac injury	368 (11.0)	101 (9.0)	267 (12.0)	0.012
Co-infection	437 (13.0)	136 (12.2)	301 (13.5)	0.30
Shock	661 (19.7)	168 (15.0)	493 (22.1)	< 0.001
**In-hospital treatments,** ***n*** **(%)**^f^				
Antibiotics	2908 (85.8)	941 (84.3)	1967 (86.5)	0.08
Antivirals (including hydroxychloroquine)	2019 (59.6)	583 (52.2)	1436 (63.2)	< 0.001
Steroids	1293 (38.2)	414 (37.1)	879 (38.7)	0.39
Tocilizumab	116 (3.9)	15 (1.5)	101 (5.1)	< 0.001

^a^Missing data: *n* = 52 (1.5%)

^b^Missing data: *n* = 628 (18%, time to hospital admission); *n* = 465 (13%, time to death); 747 (21%, time to testing)

^c^Missing data: *n* = 64 (1.8%, time to death)

^d^Missing data: *n* = 265 (7.5%)

^e^Missing data, *n* = 166 (4.7%)

^f^Missing data, *n* = 128 (3.6%)

**Fig. 1:**
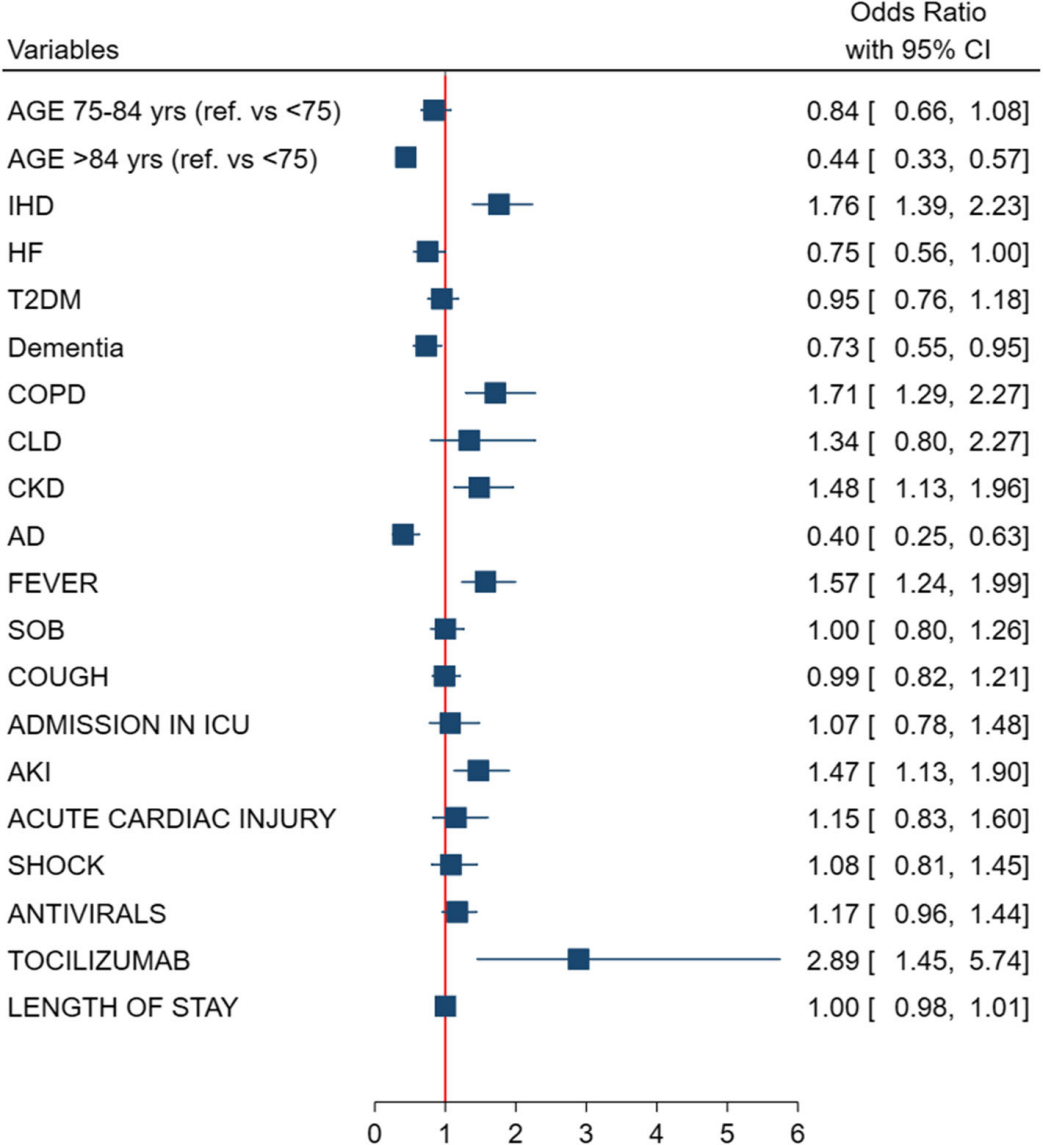
Adjusted multivariable model of clinical phenotype independently associated to being a man dying with COVID-19

To understand the complexity and the intersection of the clinical features of individuals dying from COVID-19 according to sex, a multivariable analysis adjusted for age, comorbidities, symptoms at onset, treatment received, in-hospital complications, and length of stay was performed (Fig. 1). Older age, dementia, and autoimmune disease were the specific characteristics of women dying from COVID-19, while IHD, COPD, and CKD were significantly more commonly observed in men. In addition, men were more often symptomatic with fever, were more commonly experienced AKI, and were more often treated with tocilizumab (Fig. 1).

## Discussion

In the present analysis, the COVID-19-related mortality in men was characterized by a specific clinical manifestations and transitions of care, as compared with women. Although the burden of multimorbidity was uniformly high in this older population, the distribution of diseases was sex-specific with men being more likely affected by IHD, COPD, and CKD, while women by dementia and autoimmune diseases. Fever as a symptom of onset and the occurrence of AKI were clinical features of COVID-19 dying found more often in men compared with women.

The importance of including sex- and gender-specific factors in clinical research for a more effective response to the COVID-19 pandemic has been highlighted in the past months [1–3]. Collecting large-scale sex-disaggregated data is essential for understanding the risk factors of poor outcomes and health inequities and for ultimately guiding the COVID-19 management in the future, especially now that the social restrictions have been lessened [3]. In light of this, the present analysis responded to this call for sex-informed research in fighting the pandemic providing some novel insights. The male-female health-survival paradox is a well-described condition. Women have a longer life expectancy as compared with age-matched men despite being frailer and having a higher burden of comorbidities [18, 19]. The combination of biological, behavioral, and social factors stands behind this paradox [20–22]. The disproportionate death rate in men might be partially explained by a different contribution of chronic diseases and behaviors among sexes but data are conflicting [22, 23]. In the context of the pandemic, this study extends the prior evidence that multimorbidity is highly prevalent among non-survivors [24]. Among comorbidities associated with adverse outcomes, IHD, COPD, and CKD have been reported in Italian-, Chinese-, and American-based analysis [24–28]. In our cohort, these comorbidities were more frequently observed in deceased men than women. These conditions tend to be more burdensome among men globally [29]. Of note, although women are less impacted by the pandemic, women dying of COVID-19 more often suffer from dementia and autoimmune diseases. Both diseases are known to be more prevalent among women [30, 31] and are clinical factors that might influence the occurrence of adverse events in women. A debate is ongoing on the interplay between SARS-CoV-2 infection and the impairment of the immune system typical of autoimmune diseases [32]. A recent registry-based analysis of 4842 COVID-19 Danish hospitalized patients with COVID-19 reported an excess risk (on average more than 50%) of adverse outcomes, including mortality and ICU admission in men versus women regardless of age and comorbidities, questioning the central role of advanced age and multi-morbidities in explaining the male predominance [33]. Notably, the age of the Danish cohort was on average lower and women were younger than men across all age ranges as the opposite occurred in our cohort of hospitalized deceased individuals. Moreover, in the Danish analysis, dementia was not reported and no information was provided on the severity of comorbidities at the baseline. Sex-specific detailed data are needed to better understand if the weighted impact of different comorbidities among men and women can be considered to tailor preventive strategies.

A clinical difference between men and women with COVID-19 also was observed during their in-hospital stay. Sex-specific data on the percentage of patients with COVID-19 admitted to ICU have been highlighted by the Global Health 50/50 Initiative [10]. Across 11 countries with available data on ICU admission by sex, men are more frequently admitted than women in a proportion of cases that ranges from 61% in Chile up to 74% in Norway, suggesting that an aggressive treatment strategy was reserved to most severe cases which were more prevalent in men than women. Moreover, the shortage of ICU beds which was faced by countries at the beginning of the pandemic might explain such findings. In a case series of critically ill patients with COVID-19 who required admission to the ICU in Italy, the majority were older men [34]. In our cohort of deceased individuals, male sex was not independently associated with a higher likelihood of admission in ICU in a model adjusted for confounders. Further analyses are warranted to explore the interaction of sex and age in the likelihood of being admitted in ICU and how their intersection may have an impact on major clinical outcomes.

The in-hospital stay of individuals with severe COVID-19 also identified different susceptibility to complications by sex. Beyond ARDS, other common complications in deceased patients are myocardial injury, liver or kidney injury, and shock with multi-organ dysfunction [35]. So far, no sex-disaggregated data on the rate and type of in-hospital complications have been provided. Despite knowing that men dying with COVID-19 more commonly experienced acute cardiovascular and renal complications, only the occurrence of acute kidney injury was higher in men relative to women in the multivariate adjusted model. In retrospective US-based studies, 36.6 to 78% of hospitalized patients with COVID-19 developed AKI [36, 37]. Although the paucity of data regarding the clinical characteristics of AKI in patients with COVID-19, mechanisms underlying kidney involvement in COVID-19 and the impact on survival [38] seem to be related with a direct virus-mediated or cytokine storm-mediated injury, angiotensin II pathway activation, dysregulation of innate immune system and coagulation, all effects that interact with common, and known risk factors for AKI [39]. Notably, we observed not only a higher male susceptibility to acute renal injury among deceased individuals but also that such complication was not influenced by the different pattern of comorbidities at baseline. As the pandemic unfolds, the present sex-stratified analysis can contribute to provide data on addressing questions such as whether men require additional surveillance during their in-hospital stay or early intensive tailored interventions as compared with women.

Given the similar severity of the disease (i.e., all individuals who died from COVID-19), it is noteworthy that men had a different clinical presentation than women at hospital admission. Men more often presented with the typical acute respiratory illness, with fever being a distinguishing feature. Fever is pivotal in the response to infection and the pyrogenic cytokine interleukin 6 (IL-6) has multilevel effects on the immune system [40]. In the multivariate model, fever was associated to male sex independently by other confounders: the immune response to COVID-19 and the levels of IL-6, that are associated with worse outcomes [4, 5] might be more pronounced in men, as documented by fever, and underpin the more common adverse outcomes in men than women. Despite a more typical acute respiratory illness in men, a slight delay in seeking care and later nasopharyngeal testing were observed. This finding is not surprising and may represent gender differences. The symptoms and the care seeking behavior of women differ from men in various clinical settings [41]. In fact, it is not uncommon that women seek care more than men experiencing differently symptoms as illustrated by the scenario of ischemic heart disease [42].

Finally, we found that men hospitalized for COVID-19 received more experimental therapies such as antivirals and tocilizumab than women. The reasons behind the different approach to care is not clear and difficult to explain based on the available data, most likely related to the more severe clinical progression of the disease in men. Severe COVID-19 was the first setting in which these therapies have been utilized from the pandemic onset. Of note, we do believe that even more in the context of a pandemic, treatment choices should be guided by testing the efficacy and safety of drugs in randomized controlled trials with an adequate participation of women [43, 44].

### Limitations

Our study findings should be interpreted considering the following potential limitations. The study is based on a retrospective analysis of chart reviews of only deceased individuals that were screened for main variables of interest, resulting in some missingness. We cannot ascertain the severity of the baseline comorbidities that might have played a role in the lethal outcome; however, we could only analyze sex differences between deceased people, a comparison between survivors and no-survivors would answer that question. In performing a retrospective analysis, a selection bias might have occurred; however, we minimized it by including a random sample that was representative of the age, sex, and geographical distribution of overall individuals dying for COVID-19 in Italy. There might be a bias due to the different timing of the pandemic with the first medical charts less rich in information due to the overload in terms of numbers of patients managed by the healthcare providers. Furthermore, beyond biological sex, we cannot exclude the contribution of sociocultural gender in the dynamics of COVID-19 in our study population. The collection of gender-related variables, such as identity, role, relations, and institutionalized gender is very much needed as constantly noted by the scientific community [1–3, 13, 14]. A timely UK-based analysis reported how the risk of COVID-19-related death was associated with social deprivation independent of age, sex, and comorbidities, suggesting social and gender-related factors may have an important role in COVID-19 [45, 46].

Nevertheless, these results are important because they inform clinicians of the different features of the disease that they might expect in female and male patients.

### Perspective and significance

The COVID-19 outbreak is an opportunity to explore how biological sex can shape infection disease susceptibility and outcomes. Men experiencing COVID-19-related death have a different clinical manifestations and transition of care as compared with women. A sex-informed approach in clinical research should be promoted, especially during the COVID-19 pandemic, for identifying areas likely targetable to improve outcomes and the health of both men and women.

## Data Availability

The authors declare that the data supporting the findings of this study are available within the article.

## Abbreviations

IHD: Ischemic heart disease
CHF: Congestive Heart failure
T2DM: Type 2 diabetes mellitus
CLD: Chronic liver disease
CKD: Chronic kidney disease
AD: Autoimmune disease
SOB: Shortness of breath
ICU: Intensive care unit
AKI: Acute kidney injury
COPD: Chronic obstructive pulmonary disease
COVID-19: Coronavirus disease 2019
SARS: Severe acute respiratory syndrome
ARDS: Acute respiratory distress syndrome
